# Changes in the intestinal microbiota following the administration of azithromycin in a randomised placebo-controlled trial among infants in south India

**DOI:** 10.1038/s41598-017-06862-0

**Published:** 2017-08-23

**Authors:** Edward P. K. Parker, Ira Praharaj, Jacob John, Saravanakumar Puthupalayam Kaliappan, Beate Kampmann, Gagandeep Kang, Nicholas C. Grassly

**Affiliations:** 1 Department of Infectious Disease Epidemiology, Imperial College London, London, UK; 2 Christian Medical College, Vellore, Tamil Nadu India; 3 Department of Paediatrics, Imperial College London, London, UK; 4 MRC Unit The Gambia, Fajara, Gambia

## Abstract

Macrolides are among the most widely prescribed antibiotics worldwide. However, their impact on the gut’s bacterial microbiota remains uncertain. We characterised the intestinal microbiota in 6–11 month-old infants in India who received a 3-day course of azithromycin or placebo during a randomised trial of oral poliovirus vaccine immunogenicity (CTRI/2014/05/004588). In 60 infants per study arm, we sequenced the V4 region of the bacterial 16S rRNA gene in stool samples collected before and 12 days after finishing treatment. We also tested for the presence of common bacterial, viral, and eukaryotic enteropathogens in the same samples using real-time PCR in a Taqman array card (TAC) format. Azithromycin induced a modest decline in microbiota richness and a shift in taxonomic composition driven by a reduction in the relative abundance of Proteobacteria and Verrucomicrobia (specifically *Akkermansia muciniphila*). The former phylum includes pathogenic strains of *Escherichia coli* and *Campylobacter* spp. that declined in prevalence based on the TAC assay. These findings differ from previous observations among older children and adults in Europe and North America, suggesting that the effects of azithromycin on the bacterial microbiota may be specific to the age and geographic setting of its recipients.

## Introduction

The use of antibiotics continues to rise globally. Numerous concerns have been raised regarding the long-term sequelae of this trend, including threats to populations, such as the emergence of antibiotic-resistant pathogens, and threats to individuals, such as an increased risk of metabolic disorders, asthma, and inflammatory bowel disease, among others^1–3^. Perturbations of the gut’s bacterial microbiota appear to be key to the long-term effects of antibiotic exposure. In particular, studies in mice have revealed that early-life treatment with a β-lactam or macrolide may lead to accelerated growth, delayed microbiota maturation, and a diminished capacity of the microbiota to respond to changes in diet^4^.

With upwards of 8 billion individual doses administered each year, macrolides such as azithromycin and clarithromycin are among the most widely prescribed antibiotics globally^5^. In a recent cohort of 2–7 year-old children in Finland, treatment with a macrolide within the preceding 6 months was associated with a reduction in the diversity and relative maturity of the bacterial microbiota, as well as an increase in macrolide resistance genes^6^. However, few previous studies have considered the influence of macrolide exposure on the composition of the bacterial microbiota within the context of placebo-controlled clinical trials using antibiotics. Moreover, given that significant geographic variation occurs in microbiota composition^7^, the influence of a given antibiotic on the gut’s bacterial community may differ markedly from population to population.

We recently performed a randomised placebo-controlled trial in Vellore, India, to examine the impact of a 3-day course of oral azithromycin on the immune response to a subsequent dose of monovalent type 3 oral poliovirus vaccine among seronegative 6–11 month-old infants^8^. Antibiotic treatment significantly reduced the prevalence of potentially pathogenic intestinal bacteria as well as the levels of several faecal biomarkers of intestinal inflammation and permeability, including calprotectin, myeloperoxidase, and α1-antitrypsin^8^. Here, we assess the extent to which azithromycin influenced the composition of the bacterial microbiota in these infants by sequencing the V4 region of the bacterial 16S rRNA gene in DNA extracted from stool and testing for the presence of specific pathogen gene targets using PCR in a TaqMan array card (TAC) format. In addition, we report on the impact of age on microbiota composition.

## Results

### The bacterial microbiota progresses towards a more ‘adult-like’ composition between 6 and 11 months of age

We sequenced the V4 hypervariable region of the 16S rRNA gene in 120 infants, including 60 azithromycin recipients and 60 placebo recipients. For each infant, we assessed stool samples collected before and 12 days after completion of a 3-day course of treatment (study days 0 and 14, respectively). We also included samples from 40 adults cohabiting with trial participants to provide a population-specific baseline of the mature (adult) bacterial microbiota. After quality filtering, we obtained an average of 14,685 (standard deviation 2,386) sequences per sample across the 240 infant samples and 40 adult samples that were tested. These sequences spanned 820 operational taxonomic units (OTUs) with ≥97% nucleotide identity, of which 265 were present in infants and 677 were present in adults. Sequencing of amplicons from two mock communities revealed no notable biases in amplification efficiency beyond those previously identified for the V4-specific primers used in this study (Supplementary Results; Supplementary Fig. S1)^9^.

**Figure 1:**
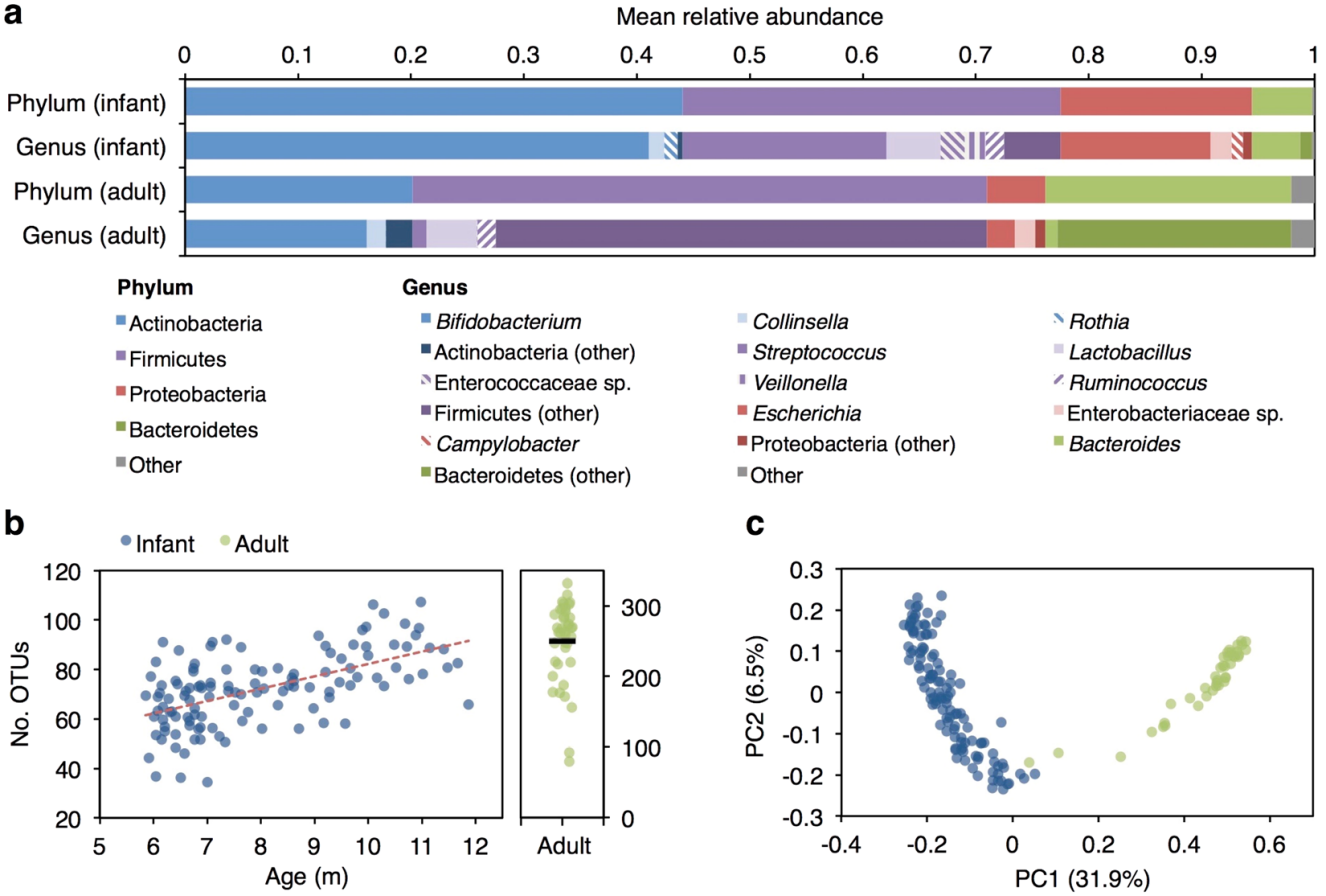
Composition of the bacterial microbiota at enrolment. (**a**) Mean relative taxon abundance at phylum and genus level among day-0 infant samples (n = 120) and adult samples (n = 40). Genera with a mean relative abundance of >1% in infants are included. For categories labeled as ‘other*’* we include genera below this abundance threshold, including 11 taxa with a mean abundance of >1% in adults. Overall, we observed 125 genera, of which 89 were present in infants and 85 were present in adults. (**b**) Number of OTUs by age. (**c**) Unweighted Unifrac distances, visualised via principal coordinates analysis, for day-0 infant samples and adult samples. Abbreviations: m, months; OTU, 97%-identity operational taxonomic unit; PC, principal coordinate.

At baseline, the bacterial microbiota was characterised by a small number of dominant taxa, evident at genus level (Fig. 1a) and OTU level – in both infants and adults, the majority of OTUs made up only a small proportion of the observed community (Supplementary Fig. S2). Combining across study arms, pre-treatment (day-0) infant samples were significantly less diverse than adult samples (72.2 ± 14.7 [mean ± standard deviation] vs 249.8 ± 57.2 for OTU count and 2.7 ± 0.5 vs 4.6 ± 0.6 for Shannon index; Student’s *t* test, p values <0.001), and clustered separately from adult samples based on Unifrac distances, which quantify the overlap in sample composition based on the proportion of the phylogenetic tree that leads to OTUs present in one but not both samples (adonis, R^2^ of 0.297 and 0.375 for unweighted distances and distances weighted by OTU abundance, respectively; p values <0.001; Fig. 1c). With increasing infant age, we observed a significant increase in day-0 OTU count (Spearman’s rho = 0.56, p < 0.001; Fig. 1b) and Shannon index (Spearman’s rho = 0.48, p < 0.001), and a decrease in mean Unifrac distance from adult samples (increasing ‘microbiota age’), reflecting a shift in composition towards a more ‘adult-like’ microbiota between 6 and 11 months of age (Spearman’s rho, −0.56 and −0.42 for unweighted and weighted Unifrac distances, respectively; p values < 0.001; Supplementary Fig. S3). The highest-ranking age-discriminatory taxa among infants, as determined by Spearman’s rho, included OTUs classified as *Faecalibacterium prausnitzii*, *Lactobacillus ruminis*, and *Dorea formicigenerans* (Supplementary Table S1) – all taxa that have been linked with microbiota maturation among infants in Bangladesh^10^.

### Azithromycin decreases microbiota diversity via depletion of Proteobacteria and *Akkermansia muciniphila*

**Figure 2:**
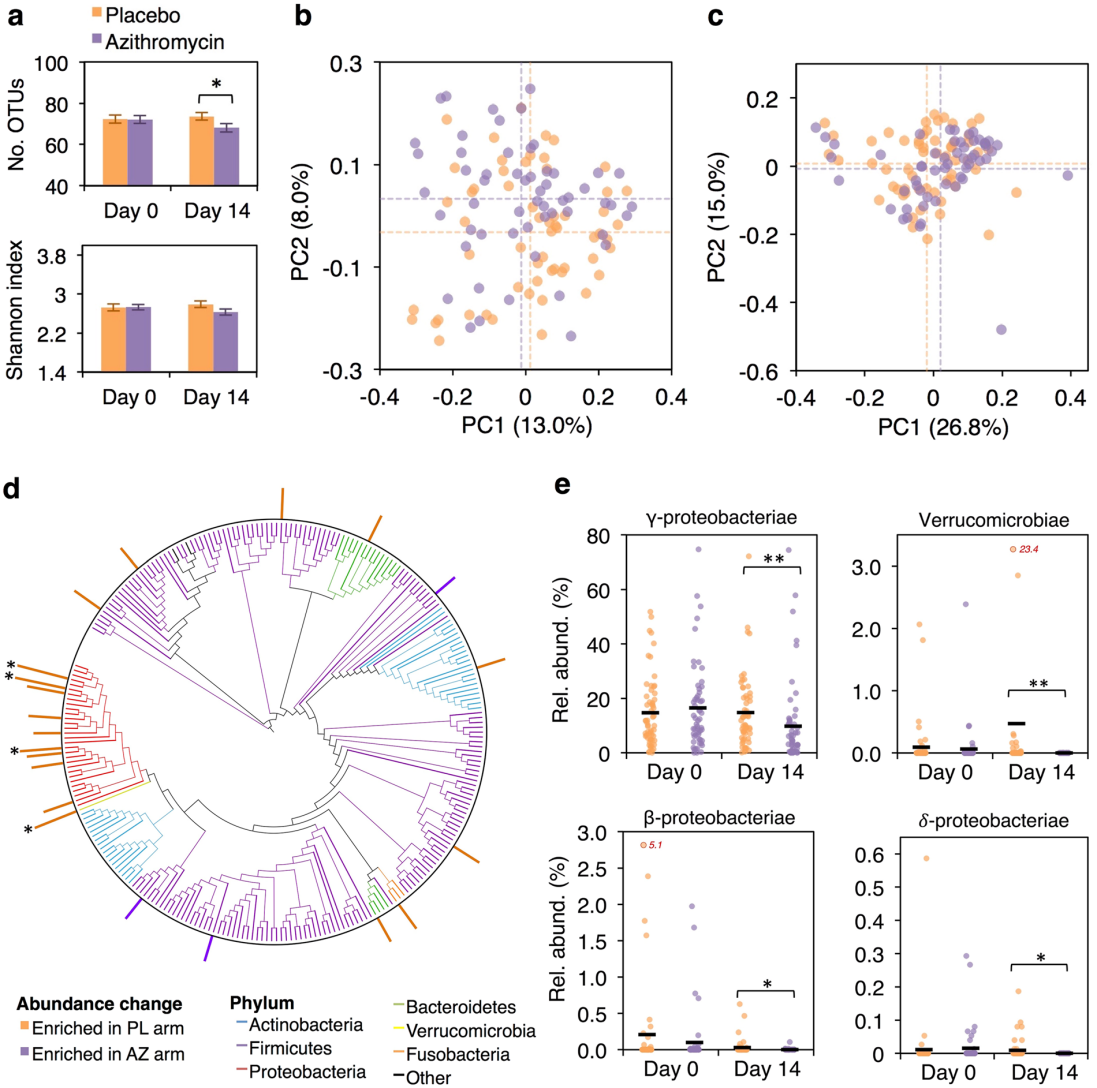
Impact of azithromycin on the bacterial microbiota. (**a**) OTU count and Shannon index (mean ± standard error) according to study arm. (**b**) Unweighted and (**c**) weighted Unifrac distances between day-14 infant samples, visualised via principal coordinates analysis. Mean values for each principal coordinate are indicated by dotted lines. (**d**) OTU-level differences in relative taxon abundance at day 14 according to study arm. Bars display p values on a negative log_10_ scale. Comparisons with a p value of <0.05 prior to FDR correction are indicated. The tree was constructed from *de novo* OTU sequences spanning the V4 region of the 16S rRNA gene. Owing to the use of a relatively short (and hypervariable) segment of the 16S rRNA gene for tree construction, phyla do not always separate into discrete lineages. (**e**) Class-level differences in taxon abundance according to study arm. Mean relative abundance values for each study arm are indicated by horizontal lines. Values beyond the scale of the y-axis are indicated in red. *P < 0.05 (after FDR correction for abundance comparisons); **FDR-corrected *P* < 0.005. Abbreviations: AZ, azithromycin; OTU, 97%-identity operational taxonomic unit; Rel. abund., relative abundance; PC, principal coordinate; PL, placebo.

Of the 120 infants included in the microbiota subset, 114 completed the trial per protocol, of whom 56 received azithromycin. Among per-protocol infants, no significant differences in infant age, microbiota diversity, microbiota age, or taxon relative abundance distinguished azithromycin from placebo recipients at day 0. After treatment (day 14), azithromycin recipients had a significantly lower OTU count than placebo recipients (68.1 ± 15.4 vs 73.6 ± 13.7; linear regression, p = 0.027 for the effect of study arm after adjusting for age; Fig. 2a). A lower Shannon index was also evident in azithromycin versus placebo recipients, albeit non-significant (2.6 ± 0.5 vs 2.8 ± 0.5; linear regression, p = 0.087). Similar trends were observed when day-0 and day-14 samples within the azithromycin arm were compared in a longitudinal analysis (mean of 72.1 ± 14.1 OTUs on day 0 versus 68.1 ± 15.4 on day 14; Wilcoxon’s test, p = 0.063; Shannon index of 2.7 ± 0.4 versus 2.6 ± 0.5, respectively; p = 0.155).

Based on Unifrac distances, we observed considerable overlap in microbiota composition between azithromycin and placebo recipients at day 14 (Fig. 2b,c). Nonetheless, treatment (study arm) accounted for a small but significant proportion of variance within the Unifrac distance matrices (adonis, R^2^ of 0.020 and 0.022 for unweighted and weighted distances, respectively; p values < 0.05). Microbiota age was comparable in azithromycin and placebo recipients at day 14 based either on unweighted Unifrac distances from adults (0.826 ± 0.042 vs 0.821 ± 0.051; linear regression, p = 0.577 for the effect of study arm after adjusting for age) or weighted distances (0.598 ± 0.041 vs 0.593 ± 0.042; linear regression, p = 0.543; Supplementary Fig. S4) – findings that were corroborated by comparing day-0 and day-14 samples among azithromycin recipients in a longitudinal analysis (Wilcoxon’s test, p values > 0.05).

The day-0 and day-14 samples collected from each infant tended to cluster together based on Unifrac distances (Wilcoxon’s test, p values < 0.001 for comparisons of within-subject versus between-subject distances; Supplementary Fig. S5). When within-subject Unifrac distances were used as a measure of microbiota stability, we observed an increase in community turnover (or decreased stability) in azithromycin compared with placebo recipients based on unweighted distances (0.485 ± 0.074 vs 0.446 ± 0.076; Wilcoxon’s test, p = 0.005; Supplementary Fig. S5B) but not on distances weighted by OTU abundance (0.230 ± 0.105 vs 0.218 ± 0.101; Wilcoxon’s test, p = 0.694; Supplementary Fig. S5D).

The observed changes in microbiota composition can be attributed primarily to decreases in the relative abundance of Proteobacteria and Verrucomicrobia in azithromycin compared with placebo recipients – at day 14, these discrepancies were evident at all taxonomic ranks tested (phylum, class, genus, and OTU; Fig. 2d,e; Supplementary Table S2). Within the phylum Verrucomicrobia, all sequences mapped to a single OTU, classified as *Akkermansia muciniphila*. These changes in taxon relative abundance after azithromycin treatment were corroborated by the comparison of day-0 and day-14 samples within a longitudinal analysis (Supplementary Table S3), but this analysis also revealed an expansion of Firmicutes (driven in part by the genus *Streptococcus*) in azithromycin recipients on day 14.

Infants were frequently exposed to antibiotics other than the study intervention before and during this trial. Among infants included in our microbiota analysis who completed the study per protocol, 28/114 (25%) took antibiotics in the month before study enrolment, while 10/114 (9%) took non-intervention antibiotics between days 0 and 14. The antibiotic regimens – recorded in 31/38 (82%) of these instances – included β-lactams (17/31 [55%]), sulphonamides (5/31 [16%]), and macrolides (3/31 [10%]), among others. Pre-enrolment antibiotic exposure was associated with a decrease in the number of OTUs present at day 0 (66.7 ± 14.9 vs 74.0 ± 13.9 in exposed and non-exposed infants, respectively; linear regression, p = 0.009 for the effect of antibiotic exposure after adjusting for age) and a corresponding decline in microbiota age based on unweighted Unifrac distances from adults, which were higher in exposed compared with non-exposed infants (0.837 ± 0.048 vs 0.814 ± 0.044; linear regression, p = 0.012). However, these discrepancies were no longer evident at day 14 (Supplementary Results; Supplementary Fig. S6).

The observed effects of azithromycin on microbiota composition (summarised in Table 1) were predominantly unchanged when infants who received non-intervention antibiotics were excluded. Effect sizes for comparisons of alpha and beta diversity at day 14 were very similar to those observed in the primary analyses (Supplementary Table S4), and a significant reduction in Proteobacteria and Verrucomicrobia abundance in azithromycin compared with placebo recipients was apparent (Wilcoxon’s test, false discovery rate [FDR]-corrected p values 0.001 and 0.002, respectively). The analyses were similarly unchanged when infants who failed to complete the study per protocol were included (data not shown).

**Table 1:** Summary of associations between azithromycin treatment and microbiota composition at day 14.

Outcome	Measure(s)	Effect
Alpha diversity	Number of OTUs/Shannon index	OTU count ~7% lower in AZ than PL arm
Beta diversity	Clustering based on unweighted/weighted Unifrac distances	Significant clustering by study arm (R^2^ of 0.02)
Taxon abundance	Phylum-, class-, genus-, and OTU-level relative abundances	Decrease in several Proteobacteria strains and *A. muc*. in AZ arm
Microbiota age	Average distance from adult samples (unweighted/weighted Unifrac)	No significant association
Microbiota stability	Distance between day-0 and day-14 samples (unweighted/weighted Unifrac)	Larger unweighted (but not weighted) distances in AZ arm
Other	Predictive accuracy of Random Forest models based on OTU abundances	Predictive accuracy of 66.7% (baseline accuracy, 50.9%)

Abbreviations: *A. muc*., *Akkermansia muciniphila*; AZ, azithromycin; OTU, 97%-identity operational taxonomic unit; PL, placebo.

### Random Forests distinguish azithromycin from placebo recipients with modest predictive accuracy

**Figure 3:**
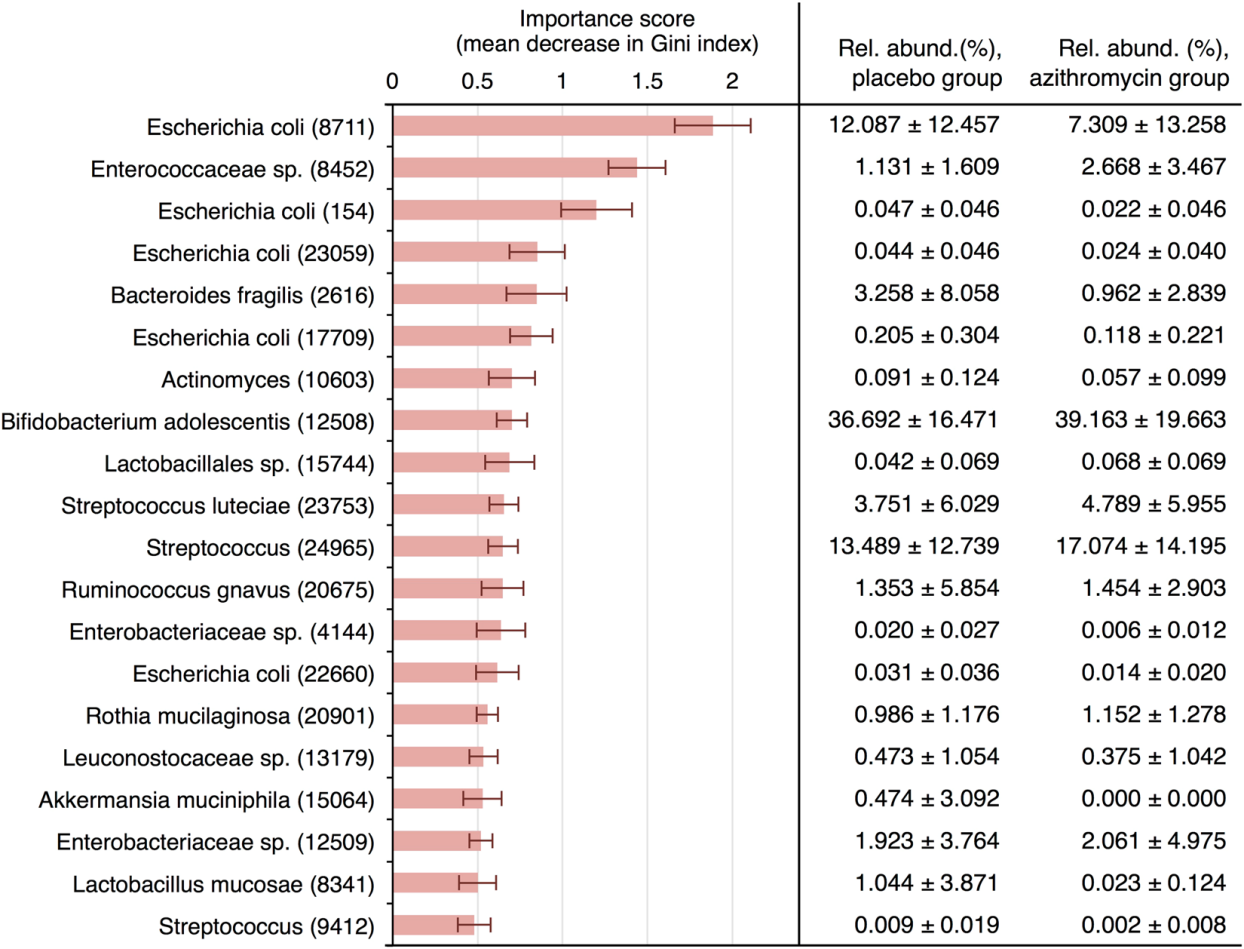
Highest ranking taxa by Random Forest importance score for prediction of study arm after treatment with azithromycin. Importance scores were calculated based on the decrease in Gini impurity index associated with inclusion of each variable in a tree. Mean importance scores (±standard deviation) were calculated across 200 cross-validation iterations of the Random Forests algorithm, with 5,000 trees per iteration.

To further elucidate the potential effects of azithromycin on microbiota composition, we used the Random Forests statistical-learning algorithm to classify infants according to study arm based on the relative abundances of OTUs on days 0 and 14. As expected, models lacked any discriminatory power at day 0 (median accuracy during cross-validation, 41.7%; interquartile range, 32.5–51.1%; baseline accuracy [wherein all infants are assigned to the majority class], 50.9%). Predictive accuracy was higher at day 14 (mean accuracy, 66.7%; interquartile range, 58.3–81.8%). This represents a rather meagre increase over baseline accuracy (50.9%), but a significant improvement in fit compared with null models in which class labels (i.e., azithromycin/placebo) were randomly assigned to the input variables (p < 0.001). OTUs with the highest importance scores for accurate prediction of study arm generally overlapped with those exhibiting depleted relative abundances following azithromycin treatment (Fig. 3; Supplementary Tables S2 and S3).

### Azithromycin has a therapeutic and prophylactic effect on bacterial enteropathogens

Asymptomatic carriage of intestinal pathogens detected by TAC was pervasive in this population. Indeed, in the study population as a whole, the proportion of infants harbouring ≥1 bacterial, viral, or eukaryotic enteropathogen was 662/704 (94%) and 636/704 (90%) at days 0 and 14, respectively. Meanwhile, diarrhoea was observed in 13/704 (2%) infants at each timepoint, and did not differ according to study arm at day 0 (7/347 [2%] vs 6/357 [2%] for azithromycin and placebo recipients, respectively; Fisher’s test, p = 0.786) or day 14 (3/347 [1%] vs 10/357 [3%]; Fisher’s test, p = 0.090). We previously demonstrated that azithromycin reduced the prevalence of bacterial enteropathogens in these infants at day 14 – an effect that was absent for viral and eukaryotic targets^8^.

To distinguish between the therapeutic versus prophylactic effects of antibiotic exposure, we assessed the impact of study arm on: (i) the proportion of individuals infected with a given enteropathogen on day 0 who retained this pathogen on day 14 (treatment); and (ii) the proportion of individuals uninfected at day 0 who became infected at day 14 (prophylaxis). Based on this approach, we found that azithromycin recipients were significantly less likely than placebo recipients to become colonised by bacterial pathogens – including enteroaggregative *Escherichia coli*, enteropathogenic *E. coli*, and *Campylobacter –* between days 0 and 14 (Table 2). Enterotoxigenic *E. coli* (ETEC) was the only common bacterial pathogen to go against this trend, newly infecting approximately 15% of infants in each study arm between days 0 and 14. Among infants infected on day 0, azithromycin consistently reduced the prevalence of bacterial enteropathogens (including ETEC) by day 14 (Table 2). Study arm did not significantly impact the rates of persistence or new infection for viral or eukaryotic enteropathogens (Fisher’s test, p values > 0.05 for all comparison).

**Table 2:** Therapeutic versus prophylactic effects of azithromycin.

Pathogen	Prevalence on day 0, n (%)	Therapy: proportion of day 0 infections retained on day 14, n/N (%)			Prophylaxis: prevalence on day 14 among infants uninfected on day 0, n/N (%)		
		Placebo	Azithromycin	p	Placebo	Azithromycin	p
≥1 Bacteria	625 (88.8)	288/317 (90.9)	209/308 (67.9)	<0.001	29/40 (72.5)	16/39 (41.0)	0.006
EAEC	514 (73.0)	215/254 (84.6)	116/260 (44.6)	<0.001	52/103 (50.5)	20/87 (23.0)	<0.001
EPEC	292 (41.5)	77/146 (52.7)	56/146 (38.4)	0.019	80/211 (37.9)	44/201 (21.9)	<0.001
ETEC	125 (17.8)	13/54 (24.1)	7/71 (9.9)	0.047	44/303 (14.5)	40/276 (14.5)	1.000
*Campylobacter*	161 (22.9)	40/85 (47.1)	12/76 (15.8)	<0.001	47/272 (17.3)	13/271 (4.8)	<0.001
*Bacteroides fragilis*	63 (8.9)	22/31 (71.0)	14/32 (43.8)	0.042	12/326 (3.7)	7/315 (2.2)	0.276
*Clostridium difficile*	19 (2.7)	5/12 (41.7)	1/7 (14.3)	0.333	4/345 (1.2)	6/340 (1.8)	0.543
*Salmonella*	19 (2.7)	1/9 (11.1)	0/10 (0.0)	0.474	3/348 (0.9)	1/337 (0.3)	0.624

Prevalence was compared according to study arm using Fisher’s exact test. Bacterial enteropathogens present in at least 2% of infants on day 0 are included. Abbreviations: EAEC, enteroaggregative *Escherichia coli*; EPEC, enteropathogenic *E. coli*; ETEC, enterotoxigenic *E. coli*.

## Discussion

Azithromycin inhibits the growth of numerous Gram-negative and Gram-positive bacteria, and is widely used to treat infections of the respiratory tract, urogenital tract, and skin^11^. Antibacterial mechanisms are varied, encompassing the inhibition of protein synthesis, the disruption of quorum sensing, and interference with adherence to host cells, among other^11, 12^. Activity extends to several common bacterial enteropathogens – in the present study, azithromycin had both a therapeutic effect (among infants infected on day 0) and a prophylactic effect (among uninfected infants) lasting for at least 12 days after the cessation of treatment. It is likely that the latter reflects, in part, the extended half-life of azithromycin in mucosal tissues (2–4 days)^13^. The extracellular release of azithromycin by macrophages and neutrophils (in which the macrolide achieves high concentrations) also appears to be dependent on the presence of bacteria at a site^14^. In spite of the significant reductions in pathogen burden observed among azithromycin recipients in this study using sensitive molecular detection methods, it is notable that the majority of infections occurred in the absence of diarrhoea and are thus of equivocal clinical significance. During a recent observational cohort study in Vellore, antibiotic treatment of diarrhoea was associated with a decrease in the median interval to a child’s next diarrhoeal episode^15^. Thus, the short-term reduction in pathogen burden observed following azithromycin treatment may not translate to a longer-term protective effect against diarrhoea.

Although the antimicrobial effects of azithromycin have been studied in relation to pathogenic bacteria, little is known regarding the impact of this drug on the bacterial microbiota as a whole, particularly during infancy. We observed a ~7% reduction in bacterial microbiota richness and a shift in composition 12 days after a 3-day course of treatment. Random Forest models based on microbiota composition were somewhat effective at distinguishing infants according to study arm after azithromycin treatment, but their predictive accuracy was surprisingly limited given the recency of antibiotic exposure. The relatively modest treatment effect that we observed may in part reflect the normalisation of the microbiota after the cessation of antibiotics, as well as the fact that OTUs in several phyla (including Actinobacteria, Bacteroidetes, and Firmicutes) were largely unaffected by azithromycin. The observed discrepancies in taxonomic composition according to study arm were driven primarily by the azithromycin-induced depletion of Proteobacteria (in particular the dominant genus *Escherichia*) and *A. muciniphila* (phylum Verrucomicrobia).

Our results differ from recent findings from Europe and North America regarding the impact of macrolides on microbiota composition. Korpela *et al*.^6^ observed a significant decrease in the relative abundance of Actinobacteria and a corresponding increase in Proteobacteria and Bacteroidetes among 2–7 year-old Finnish children exposed to azithromycin or clarithromycin within the preceding 6 months (although in agreement with our study an increased abundance of the genus *Streptococcus* in response to macrolide exposure was also reported). Meanwhile, Abeles *et al*.^16^ documented the depletion of Erysipelotrichaceae, Veillonellaceae, and Clostridiales (all members of the phylum Firmicutes) among US adults following a 3- or 7-day course of azithromycin as opposed to placebo. *A. muciniphila* is a mucin-degrading species of Gram-negative bacteria that has been suggested to play an important role in adipose tissue homeostasis^17^ and prevention of intestinal inflammation^18^. An increased abundance of this taxon has been observed following the treatment of adults with a range of non-macrolide antibiotic regimens^19^ and of mice with the veterinary macrolide tylosin^4^. However, while we observed *A. muciniphila* in 20% of azithromycin recipients on day 0, it was entirely absent from the post-treatment samples of these infants. Notably, these changes occurred in conjunction with a decrease in the levels of several faecal biomarkers of intestinal inflammation^8^.

The discord between previous findings and our own likely reflects discrepancies in trial setting and age of participants – both of which markedly alter the baseline composition of the microbiota upon which azithromycin has the potential to act – as well as time since antibiotic exposure. Indeed, the phylum Proteobacteria had a mean relative abundance of 17% at baseline in the present study, but had a low abundance (<5%) in the majority of children in the study by Korpela *et al*.^6^. This phylum also contains enteroaggregative *E. coli*, *Campylobacter*, and several of the other bacterial enteropathogens that declined in prevalence following azithromycin treatment in the present study. In addition to differences in baseline composition of the bacterial microbiota, the prevalence of antibiotic resistance genes (the ‘resistome’) may differ markedly among populations^20^. These differences may play an important role in shaping the effect of antibiotic exposure on microbiota composition.

Azithromycin did not significantly influence microbiota age, which we assessed among infant samples by measuring their mean Unifrac distance from adult samples. Previous findings regarding the impact of antibiotics on microbiota maturity have been mixed – while antibiotic exposure within the preceding 7 days did not influence relative microbiota maturity among infants in Bangladesh^10^, a decrease in microbiota maturity was apparent up to 2 years after macrolide exposure in the aforementioned Finnish cohort^6^. Similarly, early-life antibiotic exposure in mice resulted in delayed maturation of the bacterial microbiota^4^. Given that the effects of azithromycin were assessed just 12 days after the cessation of treatment, we cannot rule out a longer-term impact of antibiotic exposure on the developmental trajectory of the microbiota in the present study population.

Several limitations of this work should be acknowledged. Biases in amplification efficiency and discriminatory power arise when profiling the bacterial microbiota using amplicon sequencing^21^. Although these could have been exacerbated in the present study through the use of non-degenerate primers rather than degenerate primers that target somewhat more bacterial taxa (such as those used by Caporaso *et al*.^22^), the taxonomic distributions we obtained from mock communities of known composition were largely consistent with those previously attained using degenerate V4-specific primers^9^. Perhaps more importantly, our sequencing pipeline captures changes in the relative (rather than absolute) abundance of bacterial taxa at a given sequencing depth. We may therefore have failed to describe changes in absolute abundance of the bacterial microbiota in azithromycin recipients. As noted above, the short-term effects of azithromycin captured in our study may differ from the longer-term sequelae of antibiotic exposure^6, 23^. Finally, by focusing on changes in microbiota diversity and composition of the bacterial microbiota, we may have failed to capture changes in the functional capacity of the microbiome following azithromycin treatment, including the potential emergence of macrolide resistance^24^ and the alteration of metabolic phenotypes^4, 25^.

Overall, our findings revealed a modest effect of azithromycin on the composition of the bacterial microbiota among 6–11 month-old infants in south India. Although azithromycin brought about a decrease in microbiota diversity and a reduction in the abundance of several taxa, antibiotic treatment did not appear to influence the relative maturity of the bacterial microbiota. In addition, the bacterial taxa most affected by azithromycin differed markedly from those identified in previous studies of macrolide exposure in Europe and North America. Thus, changes in composition of the bacterial microbiota following treatment with a given antibiotic appear to be highly specific to the age and geographic setting of its recipients.

## Methods

### Study population

A full description of the design, laboratory methods, and primary outcomes of the study has previously been published^8^. Infants were randomised 1:1 to receive a 3-day course of azithromycin (administered once daily at a dose of 10 mg/kg) or placebo (comprising a syrup of matching colour and taste), starting on study day 0. A subset of 120 infants (60 per study arm) out of the first 300 enrolled in the study were selected at random for assessment of the bacterial microbiota. These infants were also assessed for the presence of multiple potentially pathogenic bacteria, viruses, and eukaryotes in stool samples via real-time PCR using TACs, as previously described^8^. The trial complied with good clinical practice guidelines and the ethical principles of the Declaration of Helsinki. Written informed consent for enrolment was obtained from a parent or caregiver. The study was approved by the Institutional Review Board of the Christian Medical College and the Drugs Controller General of India, and was registered with the Clinical Trials Registry India on 09 May 2014 (CTRI/2014/05/004588).

### Characterisation of the 16S rRNA microbiota

Full details of the laboratory and bioinformatic methods adopted in this study are provided in the Supplementary Materials. Briefly, DNA was extracted from 200 mg of stool, and the V4 region of the 16S rRNA gene subsequently amplified using primers 515F (5′-GTGCCAGCAGCCGCGGTAA-3′) and 806R (5′-GGACTACCAGGGTATCTAAT-3′). Purified PCR products were sequenced in a single Illumina MiSeq run that also included one no-template PCR control, 14 no-template extraction controls, and PCR products amplified from two mock communities obtained from BEI resources (Manassas, USA). After sequencing, reads were assembled into contigs using FLASH^26^ and analysed using QIIME (MacQIIME version 1.8.0). Contigs were quality-filtered^27^, then clustered *de novo* into OTUs with ≥97% nucleotide identity using a modified version of the ‘denovo.sh’ pipeline published by Nelson *et al*.^9^. All analyses were performed at a rarefaction depth of 7,500 sequences per sample.

### Baseline composition of the bacterial microbiota

OTU count and Shannon index were compared between day-0 infant samples and adult samples using Student’s *t* test. Unifrac distances between these samples, either weighted by taxon abundance (weighted Unifrac) or unweighted (unweighted Unifrac)^28^, were visualised using principal coordinates analysis, and cluster significance assessed using the adonis function (in the R package *vegan* ^29^) with 9,999 permutations. We used the mean Unifrac distance of each infant sample from non-cohabiting adults as an indicator of microbiota age. Two adult samples were excluded from calculations of infant microbiota age as they had substantially lower diversity than the remaining adult samples (Fig. 1a) and clustered with infant samples based on unweighted Unifrac distances (Fig. 1b). Changes in microbiota diversity, microbiota age, and OTU relative abundances with respect to infant age were assessed in day-0 samples using Spearman’s rho.

### Impact of azithromycin on the bacterial microbiota

Analyses regarding the effect of azithromycin on microbiota composition were restricted to individuals who completed the study per protocol (see Supplementary Materials for a summary of protocol deviations). Infant age at day 0 was compared according to study arm using Wilcoxon’s rank sum test. The effect of azithromycin on OTU count, Shannon index, and microbiota age (as continuous dependent variables) was assessed in cross-sectional analyses at days 0 and 14 via linear regression (with study arm as a categorical explanatory variable and age included as a covariate). We also performed longitudinal analyses to compare these metrics between day-0 and day-14 samples in each study arm using Wilcoxon’s signed rank test for paired data. Unifrac distances between infant samples were visualised using principal coordinates analysis and cluster significance assessed using adonis. Age was included prior to study arm when implementing adonis and significance determined based on sequential sums of squares.

Phylum-, class-, genus-, and OTU-level discrepancies in taxon relative abundance were compared between study arms using Wilcoxon’s rank sum test (cross-sectional analyses), and between day-0 and day-14 samples in each arm using Wilcoxon’s signed rank test (longitudinal analyses). Taxa were assessed if they were present in at least 2% of samples being compared. To account for multiple comparisons, p values were adjusted by Benjamini–Hochberg FDR correction^30^, applied separately at each taxonomic rank for a given comparison. OTUs associated with study arm were visualised using Interactive Tree of Life^31^. Within-subject distances (between samples collected from the same infant at days 0 and 14) were compared with between-subject Unifrac distances (comprising the average Unifrac distance of each day-0 sample from the day-14 samples collected from other infants) using Wilcoxon’s rank sum test. The effect of azithromycin on within-subject Unifrac distances was assessed using Wilcoxon’s rank sum test. For comparisons of taxon abundance, we report on any associations with an FDR-corrected p value of <0.15. For all other comparisons, p values of <0.05 were considered statistically significant.

### Impact of non-intervention antibiotics

Exposure to antibiotics in the month before enrolment was recorded on day 0 and use of non-intervention antibiotic post-enrolment was recorded on day 14 through interview of the caregiver by the study physician. The name of the antibiotic was recorded where possible from the used medicine container or prescription. The effect of pre-enrolment antibiotic exposure on OTU count, Shannon index, microbiota age, sample clustering based on Unifrac distances, and taxon relative abundances was assessed on days 0 and 14 using the methods described above for cross-sectional analyses of study arm (see Supplementary Materials for additional details). The potential association between non-intervention antibiotic use and study arm was assessed using Fisher’s exact test.

### Random Forests

Random Forest models were implemented in the R package *randomForest* to discriminate infants according to study arm based on the composition of their bacterial microbiota^32^. A table of OTU relative abundances served as input for the models. Separate analyses were carried out for day-0 and day-14 samples. For each analysis, out-of-bag error rates and variable importance scores (based on Gini index) were determined across 20 cycles of 10-fold cross-validation (200 iterations in total), with 5,000 trees per iteration and all other parameters at their default values. Cross-validation was implemented in the R package *crossval* ^33^. Significance of the model fit was assessed by generating 999 null models in which study arm labels were randomly assigned to the input variables and determining the proportion of null models with an out-of-bag error rate smaller than the median error rate of the fitted models.

### Enteropathogen prevalence

We assessed the impact of study arm on the prevalence of novel infections (absent on day 0, present on day 14) or persistent infections (present on days 0 and 14) based on TAC data, and concurrent diarrhoea, using Fisher’s exact test.

### Sensitivity analyses

We performed sensitivity analyses in which: (i) the effect of azithromycin on microbiota composition was assessed among infants with no exposure to non-intervention antibiotics; and (ii) comparisons were performed for all infants providing stool samples without restriction to per-protocol infants. In each case, we compared OTU count, Shannon index, microbiota age, and phylum-level taxon relative abundances according to study arm in day-14 samples using the cross-sectional analysis methods described above.

### Data availability

16S rRNA sequences generated from faecal samples have been deposited in the European Nucleotide Archive (accession number PRJEB20773). An OTU table obtained after sequence assembly, quality filtering, chimera checking, taxonomic assignment, and minimum abundance filtering is provided in biom format in the Supplementary Materials, as is a file containing relevant metadata.

## Electronic supplementary material

Supplementary Information
