# Supplementary material for: Changes in the intestinal microbiota following the administration of azithromycin in a randomised placebo-controlled trial among infants in south India
Source: Sci Rep. 2017 Aug 23;7:9168. doi: 10.1038/s41598-017-06862-0 (PMC5569098; doi:10.1038/s41598-017-06862-0)
Supplement: Supplementary file 1 — Supplementary Information [file 41598_2017_6862_MOESM1_ESM.zip › 6862 Supplementary Tables and figures.pdf]

Supplementary Tables and Figures

Changes in the intestinal microbiota following the administration of azithromycin in a randomized placebo-controlled trial among infants in south India

Edward PK Parker, Ira Praharaj, Jacob John, Saravanakumar Puthupalayam Kaliappan, Beate Kampmann, Gagandeep Kang, Nicholas C Grassly

Supplementary Table 1. Top 20 age-discriminatory operational taxonomic units.

Taxonomy	OTU ID	Prevalence (%)		Relative abundance (mean % ± s.d.)		rho	p
		<8 m (n = 70)	≥8 m (n = 50)	<8 m (n = 70)	≥8 m (n = 50)		
Faecalibacterium prausnitzii	9677	25.7	66.0	0.057 ± 0.154	0.844 ± 2.054	0.49	1.16E-08
Lactobacillus ruminis	12511	30.0	62.0	0.871 ± 5.516	2.693 ± 5.754	0.44	5.12E-07
Ruminococcaceae sp.	19836	8.6	44.0	0.045 ± 0.289	0.067 ± 0.129	0.44	4.83E-07
Faecalibacterium	13285	4.3	30.0	0.001 ± 0.003	0.021 ± 0.054	0.42	1.75E-06
Dorea formicigenerans	13285	17.1	52.0	0.096 ± 0.360	0.207 ± 0.382	0.41	3.00E-06
Faecalibacterium	5200	8.6	32.0	0.003 ± 0.010	0.017 ± 0.043	0.37	3.26E-05
Coprococcus	18159	7.1	36.0	0.062 ± 0.370	0.257 ± 0.709	0.37	2.84E-05
Ruminococcus gnavus	9067	11.4	38.0	0.120 ± 0.566	0.615 ± 1.532	0.35	8.86E-05
Blautia	15230	7.1	34.0	0.093 ± 0.495	0.113 ± 0.301	0.35	9.28E-05
Lachnospiraceae sp.	15571	7.1	28.0	0.026 ± 0.135	0.042 ± 0.122	0.35	0.0001
Blautia	3067	17.1	44.0	0.022 ± 0.068	0.466 ± 1.135	0.34	0.0001
Lactobacillus	2346	7.1	30.0	0.011 ± 0.074	0.035 ± 0.128	0.33	0.0002
Lactobacillus	2549	7.1	28.0	0.038 ± 0.282	0.054 ± 0.120	0.33	0.0003
Streptococcus infantis	5074	61.4	34.0	0.022 ± 0.031	0.008 ± 0.015	-0.33	0.0003
Bifidobacterium adolescentis	19111	7.1	28.0	0.001 ± 0.005	0.016 ± 0.039	0.32	0.0004
Ruminococcus	4023	5.7	26.0	0.155 ± 1.033	0.379 ± 2.078	0.31	0.0005
Lachnospiraceae sp.	23320	4.3	20.0	0.004 ± 0.020	0.015 ± 0.048	0.31	0.0005
Leuconostocaceae sp.	15235	32.9	58.0	0.018 ± 0.051	0.027 ± 0.050	0.30	0.0008
Streptococcus	23667	11.4	32.0	0.004 ± 0.012	0.014 ± 0.029	0.30	0.0009
Lactobacillus ruminis	19280	8.6	28.0	0.005 ± 0.018	0.015 ± 0.037	0.30	0.0010

OTUs that showed monotonic changes in relative abundance with increasing age were identified based on Spearman's rho. The top 20 age-discriminatory taxa are displayed above for pre-treatment (day-0) samples, combining across study arms. Abbreviations: m, months; OTU, 97%-identity operational taxonomic unit.

Supplementary Table 2. Influence of azithromycin on taxon abundance (cross-sectional analysis).

Taxonomic classification	OTU ID	Prevalence (%) PL (n = 58)	Prevalence (%) AZ (n = 56)	Relative abundance (mean % ± s.d.) PL (n = 58)	Relative abundance (mean % ± s.d.) AZ (n = 56)	p	FDR p
**Phylum (all)**							
Proteobacteria	-	100.0	98.2	15.889 ± 13.207	10.200 ± 15.401	0.0001	0.0008
Verrucomicrobia	-	20.7	0.0	0.474 ± 3.092	0.000 ± 0.000	0.0004	0.0012
Fusobacteria	-	37.9	21.4	0.151 ± 0.856	0.745 ± 4.609	0.0509	0.1189
Bacteroidetes	-	89.7	83.9	5.163 ± 10.268	3.497 ± 7.518	0.1075	0.1882
Actinobacteria	-	100.0	100.0	41.017 ± 17.551	44.353 ± 20.885	0.2114	0.2959
Firmicutes	-	100.0	100.0	37.275 ± 19.016	41.177 ± 20.596	0.3268	0.3813
Other	-	79.3	80.4	0.031 ± 0.031	0.028 ± 0.025	0.7529	0.7529
**Class (FDR p <0.15)**							
Gammaproteobacteria	-	100.0	96.4	14.801 ± 13.270	9.812 ± 15.365	0.0002	0.0025
Verrucomicrobiae	-	20.7	0.0	0.474 ± 3.092	0.000 ± 0.000	0.0004	0.0025
Deltaproteobacteria	-	13.8	0.0	0.009 ± 0.031	0.000 ± 0.000	0.0042	0.0197
Betaproteobacteria	-	24.1	7.1	0.030 ± 0.106	0.003 ± 0.014	0.0096	0.0335
Fusobacteria	-	37.9	21.4	0.151 ± 0.856	0.745 ± 4.609	0.0509	0.1280
Erysipelotrichi	-	62.1	76.8	0.242 ± 0.607	0.527 ± 1.128	0.0548	0.1280
**Genus (FDR p <0.15)**							
Escherichia	-	100.0	96.4	12.459 ± 12.682	7.506 ± 13.453	8.3E-05	0.0066
Akkermansia	-	20.7	0.0	0.474 ± 3.092	0.000 ± 0.000	0.0004	0.0143
Bilophila	-	13.8	0.0	0.009 ± 0.031	0.000 ± 0.000	0.0042	0.0933
Fusobacterium	-	20.7	3.6	0.020 ± 0.095	0.000 ± 0.003	0.0047	0.0933
Sutterella	-	12.1	0.0	0.018 ± 0.088	0.000 ± 0.000	0.0077	0.1225
Peptostreptococcus	-	15.5	1.8	0.345 ± 1.839	0.000 ± 0.002	0.0092	0.1225
**OTU (FDR p <0.15)**							
Escherichia coli	8711	100.0	96.4	12.087 ± 12.457	7.309 ± 13.258	0.0001	0.0133
Escherichia coli	154	74.1	42.9	0.047 ± 0.046	0.022 ± 0.046	0.0001	0.0133
Akkermansia muciniphila	15064	20.7	0.0	0.474 ± 3.092	0.000 ± 0.000	0.0004	0.0295
Enterobacteriaceae sp.	4144	58.6	30.4	0.020 ± 0.027	0.006 ± 0.012	0.0005	0.0329
Escherichia coli	23059	75.9	53.6	0.044 ± 0.046	0.024 ± 0.040	0.0012	0.0582
Escherichia coli	22660	67.2	42.9	0.031 ± 0.036	0.014 ± 0.020	0.0019	0.0796
Campylobacter	21529	19.0	1.8	1.037 ± 3.858	0.001 ± 0.005	0.0029	0.0868
Escherichia coli	17709	91.4	69.6	0.205 ± 0.304	0.118 ± 0.221	0.0035	0.0868
Streptococcus	9412	27.6	7.1	0.009 ± 0.019	0.002 ± 0.008	0.0036	0.0868
Lactobacillus mucosae	8341	34.5	12.5	1.044 ± 3.871	0.023 ± 0.124	0.0038	0.0868
Bilophila	8458	13.8	0.0	0.009 ± 0.031	0.000 ± 0.000	0.0042	0.0868
Megasphaera	6777	13.8	0.0	0.007 ± 0.021	0.000 ± 0.000	0.0042	0.0868
Fusobacterium	13808	20.7	3.6	0.020 ± 0.095	0.000 ± 0.002	0.0047	0.0868
Bifidobacterium	19019	39.7	14.3	0.010 ± 0.017	0.005 ± 0.013	0.0049	0.0868
Bacteroides fragilis	2616	75.9	62.5	3.258 ± 8.058	0.962 ± 2.839	0.0059	0.0969
Sutterella	23966	12.1	0.0	0.018 ± 0.088	0.000 ± 0.000	0.0077	0.1129
Parabacteroides gordonii	22351	12.1	0.0	0.017 ± 0.092	0.000 ± 0.000	0.0077	0.1129
Dorea	17630	6.9	25.0	0.028 ± 0.190	0.042 ± 0.127	0.0088	0.1199
Peptostreptococcus	18269	15.5	1.8	0.345 ± 1.839	0.000 ± 0.002	0.0092	0.1199
Lactobacillales sp.	15744	70.7	82.1	0.042 ± 0.069	0.068 ± 0.069	0.0105	0.1261
Clostridiaceae	3647	13.8	33.9	0.005 ± 0.015	0.043 ± 0.120	0.0107	0.1261

No phylum-, class-, or OTU-level differences distinguished azithromycin from placebo recipients at enrolment (FDR-corrected *P* values >0.05). Abbreviations: AZ, azithromycin arm; FDR, adjusted by Benjamini–Hochberg false discovery rate correction; OTU, 97%-identity operational taxonomic unit; PL, placebo arm.

Supplementary Table 3. Influence of azithromycin on taxon abundance (longitudinal analysis).

Taxonomic classification	OTU ID	Prevalence (%)		Relative abundance (mean % ± s.d.)		p	FDR p
		Day 0 (n = 56)	Day 14 (n = 56)	Day 0 (n = 56)	Day 14 (n = 56)		
**Phylum (all)**							
Proteobacteria	-	100.0	98.2	17.658 ± 15.636	10.200 ± 15.401	0.0004	0.0025
Verrucomicrobia	-	19.6	0.0	0.065 ± 0.327	0.000 ± 0.000	0.0038	0.0132
Firmicutes	-	100.0	100.0	33.742 ± 18.068	41.177 ± 20.596	0.0107	0.0249
Fusobacteria	-	28.6	21.4	0.160 ± 0.670	0.745 ± 4.609	0.1520	0.2212
Bacteroidetes	-	87.5	83.9	4.647 ± 8.438	3.497 ± 7.518	0.1580	0.2212
Actinobacteria	-	100.0	100.0	43.670 ± 18.694	44.353 ± 20.885	0.7288	0.7792
Other	-	76.8	80.4	0.034 ± 0.038	0.028 ± 0.025	0.7792	0.7792
**Class (FDR p <0.15)**							
Gammaproteobacteria	-	100.0	96.4	16.485 ± 15.712	9.812 ± 15.365	0.0005	0.0073
Betaproteobacteria	-	25.0	7.1	0.099 ± 0.365	0.003 ± 0.014	0.0021	0.0149
Verrucomicrobiae	-	19.6	0.0	0.065 ± 0.327	0.000 ± 0.000	0.0038	0.0176
Deltaproteobacteria	-	14.3	0.0	0.016 ± 0.054	0.000 ± 0.000	0.0141	0.0495
Bacilli	-	100.0	100.0	27.210 ± 17.717	33.07 ± 19.255	0.0641	0.1480
Epsilonproteobacteria	-	23.2	10.7	1.034 ± 3.981	0.384 ± 1.793	0.0712	0.1480
Erysipelotrichi	-	53.6	76.8	0.534 ± 1.632	0.527 ± 1.128	0.0740	0.1480
**Genus (FDR p <0.15)**							
Escherichia	-	100.0	96.4	13.737 ± 14.447	7.506 ± 13.453	0.0002	0.0123
Sutterella	-	21.4	0.0	0.073 ± 0.343	0.000 ± 0.000	0.0022	0.0869
Akkermansia	-	19.6	0.0	0.065 ± 0.327	0.000 ± 0.000	0.0038	0.0884
Campylobacter	-	21.4	7.1	1.034 ± 3.981	0.210 ± 1.564	0.0062	0.0884
Fusobacterium	-	17.9	3.6	0.007 ± 0.017	0.000 ± 0.003	0.0065	0.0884
Proteus	-	19.6	3.6	0.112 ± 0.566	0.001 ± 0.005	0.0067	0.0884
Pasteurellaceae sp.	-	53.6	42.9	0.612 ± 1.458	0.101 ± 0.272	0.0103	0.1165
Bilophila	-	14.3	0.0	0.016 ± 0.054	0.000 ± 0.000	0.0141	0.1195
Alloiococcus	-	25.0	8.9	0.010 ± 0.023	0.002 ± 0.006	0.0144	0.1195
Streptococcus	-	100.0	100.0	19.842 ± 14.387	27.144 ± 18.671	0.0159	0.1195
Clostridium	-	46.4	67.9	0.135 ± 0.351	0.314 ± 0.961	0.0167	0.1195
Bacteroides	-	76.8	73.2	4.252 ± 8.444	1.488 ± 3.389	0.0182	0.1195
Haemophilus	-	14.3	5.4	0.012 ± 0.045	0.001 ± 0.003	0.0198	0.1201
**OTU (FDR p <0.15)**							
Escherichia coli	8711	100.0	96.4	13.419 ± 14.266	7.309 ± 13.258	0.0002	0.0410
Escherichia coli	22660	75.0	42.9	0.034 ± 0.036	0.014 ± 0.020	0.0005	0.0615
Escherichia coli	154	76.8	42.9	0.048 ± 0.057	0.022 ± 0.046	0.0013	0.1047
Sutterella	23966	21.4	0.0	0.073 ± 0.343	0.000 ± 0.000	0.0022	0.1177
Enterobacteriaceae sp.	4144	48.2	30.4	0.016 ± 0.022	0.006 ± 0.012	0.0025	0.1177
Bacteroides fragilis	2616	69.6	62.5	3.584 ± 7.991	0.962 ± 2.839	0.0031	0.1214
Akkermansia muciniphila	15064	19.6	0.0	0.065 ± 0.327	0.000 ± 0.000	0.0038	0.1282

No phylum-, class-, genus-, or OTU-level differences distinguished day-0 and day-14 samples in the placebo arm (FDR-corrected Wilcoxon's p values >0.15). Abbreviations: AZ, azithromycin arm; FDR, adjusted by Benjamini–Hochberg false discovery rate correction; OTU, 97%-identity operational taxonomic unit; PL, placebo arm.

Supplementary Table 4. Influence of pre-enrolment antibiotic exposure on taxon abundance.

Taxonomic classification	OTU ID	Prevalence (%) AB- (n = 86)	Prevalence (%) AB+ (n = 28)	Relative abundance (mean % ± s.d.) AB- (n = 86)	Relative abundance (mean % ± s.d.) AB+ (n = 28)	p	FDR p
**Day 0**							
**Phylum (all)**							
Bacteroidetes	-	94.2	78.6	6.202 ± 10.499	2.471 ± 4.960	0.0449	0.3140
Verrucomicrobia	-	20.9	14.3	0.104 ± 0.395	0.005 ± 0.018	0.3249	0.7087
Fusobacteria	-	30.2	21.4	0.111 ± 0.545	0.010 ± 0.024	0.3871	0.7087
Proteobacteria	-	100.0	100.0	16.405 ± 14.688	18.259 ± 13.842	0.4050	0.7087
Firmicutes	-	100.0	100.0	33.193 ± 17.86	35.737 ± 20.474	0.5916	0.8283
Other	-	77.9	82.1	0.033 ± 0.032	0.030 ± 0.029	0.8047	0.8797
Actinobacteria	-	100.0	100.0	43.936 ± 18.753	43.487 ± 18.375	0.8797	0.8797
**Class (FDR p <0.15)**							
Coriobacteriia	-	95.3	71.4	1.882 ± 3.075	1.401 ± 3.438	0.0059	0.0824
**Genus (FDR p <0.15)**							
Atopobium	-	62.8	21.4	0.049 ± 0.081	0.020 ± 0.062	0.0010	0.0766
Actinomyces	-	76.7	57.1	0.091 ± 0.130	0.039 ± 0.077	0.0022	0.0874
**OTU (FDR p <0.15)**							
NA							
**Day 14**							
**Phylum (all)**							
Actinobacteria	-	100.0	100.0	45.095 ± 18.811	35.164 ± 18.959	0.0226	0.1579
Firmicutes	-	100.0	100.0	37.444 ± 18.184	44.561 ± 23.727	0.2296	0.6183
Fusobacteria	-	26.7	39.3	0.581 ± 3.778	0.018 ± 0.035	0.3080	0.6183
Proteobacteria	-	98.8	100.0	12.340 ± 13.516	15.410 ± 17.407	0.3533	0.6183
Verrucomicrobia	-	11.6	7.1	0.286 ± 2.527	0.102 ± 0.539	0.5045	0.7064
Bacteroidetes	-	86.0	89.3	4.224 ± 8.399	4.717 ± 10.878	0.7441	0.8353
Other	-	81.4	75.0	0.030 ± 0.029	0.028 ± 0.026	0.8353	0.8353
**Class (FDR p <0.15)**							
Coriobacteriia	-	94.2	75.0	2.487 ± 4.659	1.012 ± 1.940	0.0046	0.0645
**Genus (FDR p <0.15)**							
Leuconostoc	-	20.9	57.1	0.018 ± 0.094	0.072 ± 0.161	9.7E-05	0.0077
**OTU (FDR p <0.15)**							
Leuconostoc	22309	20.9	57.1	0.018 ± 0.094	0.072 ± 0.161	9.7E-05	0.0240
Bacteroides	3786	4.7	28.6	0.002 ± 0.011	0.009 ± 0.024	0.0005	0.0622

Abbreviations: AB-, not exposed to antibiotics pre-enrolment; AB+, exposed to antibiotics pre-enrolment; FDR, adjusted by Benjamini–Hochberg false discovery rate correction; NA, not applicable; OTU, 97%-identity operational taxonomic unit.

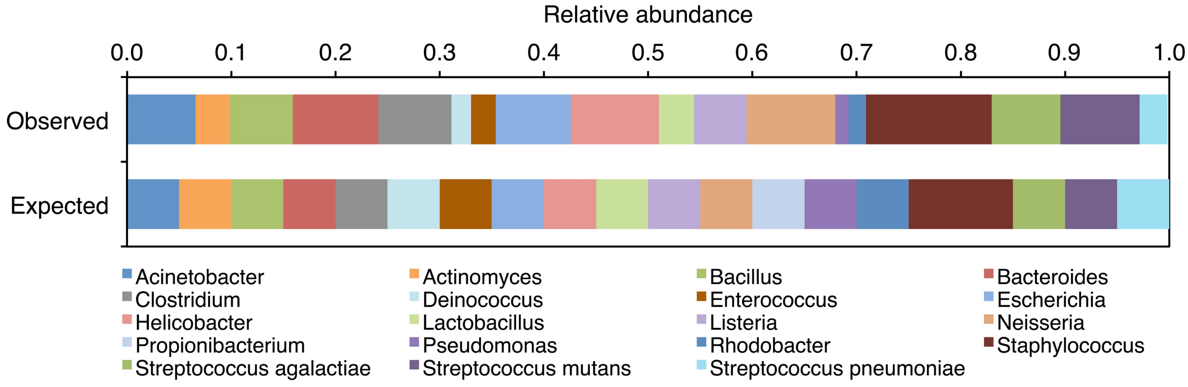

Supplementary Figure 1. Mock community composition. Data are displayed for the mock community with an even rRNA operon count per organism (HM-782D, BEI Resources).

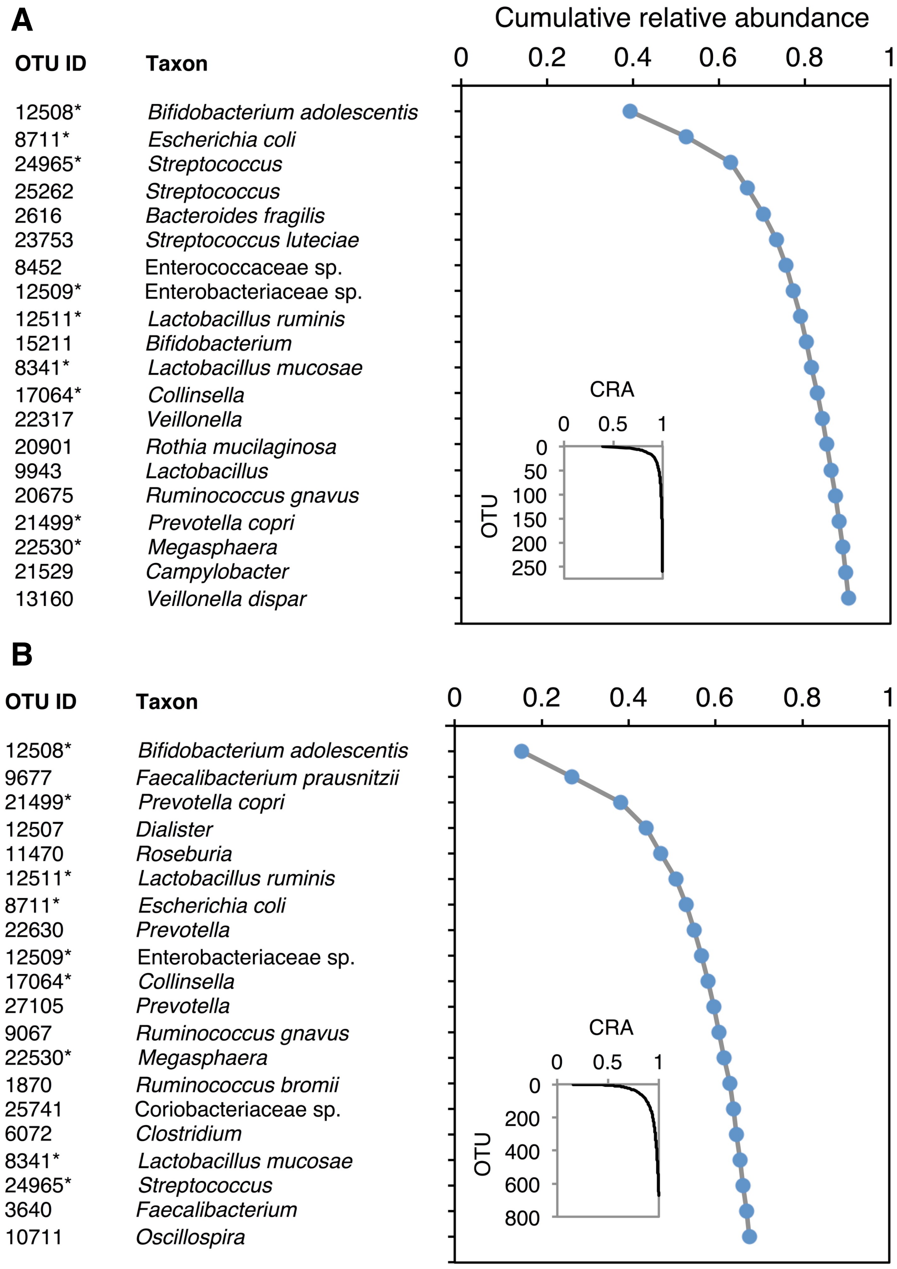

Supplementary Figure 2. Top 20 operational taxonomic units by mean relative abundance. Data are displayed for (A) day-0 infant samples and (B) adult samples. OTUs present in (A) and (B) are indicated with an asterisk. The abundance distribution of all OTUs is displayed in the inset. Abbreviations: CRA, cumulative relative abundance; OTU, 97%-identity operational taxonomic unit.

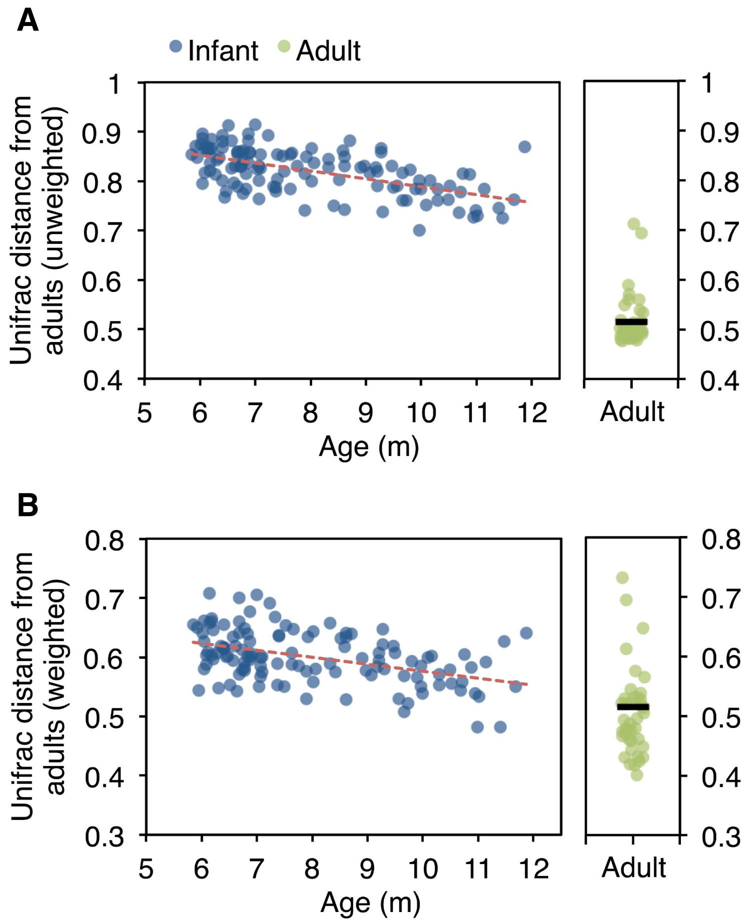

Supplementary Figure 3. Maturation of the bacterial microbiota throughout infancy. Microbiota age, as determined by mean Unifrac distance from non-cohabiting adults, is displayed by infant age for (A) unweighted Unifrac distances and (B) weighted Unifrac distances. Abbreviation: m, months.

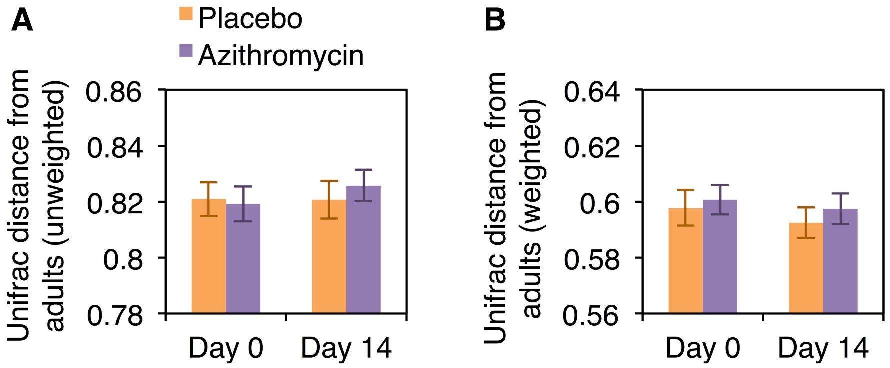

Supplementary Figure 4. Impact of azithromycin on microbiota age. Mean Unifrac distance from non-cohabiting adults is displayed by study arm for (A) unweighted Unifrac distances and (B) weighted Unifrac distances.

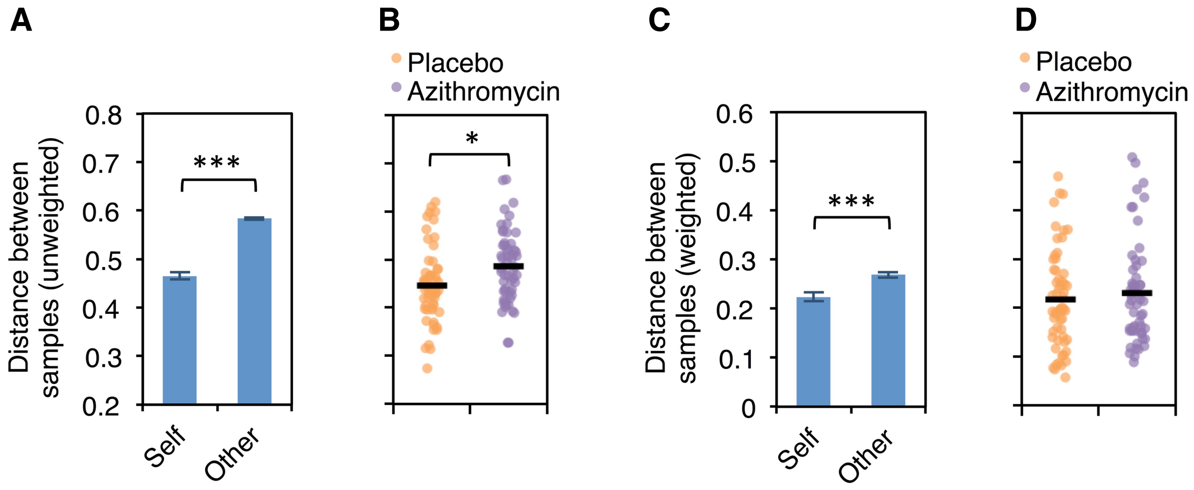

Supplementary Figure 5. Impact of azithromycin on microbiota stability. (A) Unweighted Unifrac distances (between day-0 and day-14 samples) were significantly smaller within than between individuals. Mean values (± standard error) are indicated. (B) Within-subject unweighted Unifrac distances, used as an indicator of microbiota stability, according to study arm. (C, D) Equivalent plots are shown for weighted Unifrac distances.

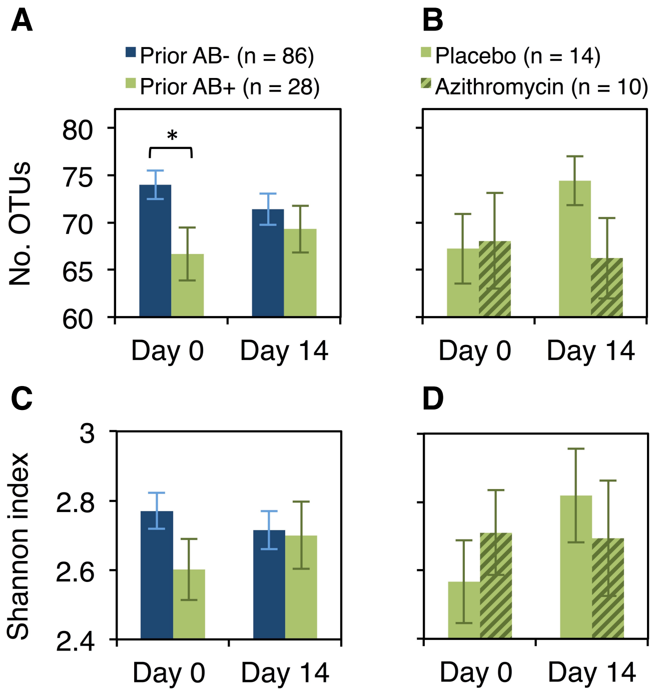

Supplementary Figure 6. Impact of non-intervention antibiotics on microbiota diversity. (A) OTU count (mean ± standard error) according to pre-enrolment antibiotic exposure status. (B) Effect of study arm on OTU count among infants exposed to antibiotics before enrolment. Infants exposed to non-intervention antibiotics between days 0 and 14 were excluded. (C, D) Equivalent plots are shown for Shannon index. Abbreviations: AB, antibiotic; OTU, 97%-identity operational taxonomic unit.
