# Supplementary material for: Changes in the intestinal microbiota following the administration of azithromycin in a randomised placebo-controlled trial among infants in south India
Source: Sci Rep. 2017 Aug 23;7:9168. doi: 10.1038/s41598-017-06862-0 (PMC5569098; doi:10.1038/s41598-017-06862-0)
Supplement: Supplementary file 1 — Supplementary Information [file 41598_2017_6862_MOESM1_ESM.zip › 6862 Supplementary methods and results.pdf]

Supplementary Methods and Results

Changes in the intestinal microbiota following the administration of azithromycin in a randomized placebo-controlled trial among infants in south India

Edward PK Parker, Ira Praharaj, Jacob John, Saravanakumar Puthupalayam Kaliappan, Beate Kampmann, Gagandeep Kang, Nicholas C Grassly

Methods

DNA extraction. DNA was extracted from 200 mg of stool using the QIAamp DNA Stool Mini Kit. The Qiagen protocol was modified to include an incubation step at 37˚C for 10 minutes with 1.67 μl of lysozyme (30 mg/ml), 13 μl of mutanolysin (11.7 U/μl), and 3 μl of lysostaphin (4.5 U/μl) – an enzyme cocktail that has been shown to improve taxonomic representativeness during studies of mock bacterial communities[1]. Incubation was carried out using a plate thermo–shaker (PST-60HL-4, Biosan) set at 250 RPM. Following incubation, we added 10 μl of proteinase K, 50 μl of sodium dodecyl sulphate (10%), and 20 μl of RNase A (1 mg/ml) to each sample prior to a second incubation step at 70˚C for 10 minutes. Samples were subsequently vortexed at high speed with 370 mg of 500-μm glass beads (added to samples at the start of the protocol) for 5 minutes. At the end of the extraction procedure, the final elution was performed using 50 μl of buffer AE. A negative control was included in each extraction batch.

Library preparation. The protocol for the preparation of the 16S rRNA gene V4 region library was adapted by Dr Anna Zekavati (Imperial Biomedical Research Centre Genomics Facility, Hammersmith Hospital, London) from a protocol developed by Balamurugan Ramadass (Department of Gastrointestinal Sciences, Christian Medical College, Vellore) and colleagues[2]. The V4 region of the 16S rRNA gene was amplified via PCR in triplicate for each sample using primers 515F (5’-GTGCCAGCAGCCGCGGTAA-3’) and 806R (5’-GGACTACCAGG-GTATCTAAT-3’). Primers also contained Illumina adapter sequences, primer pads, and linker sequences, as detailed by Caporaso et al[3]. Each reverse primer contained a unique 12-bp Golay barcode to enable multiplexed sequencing. The reaction mixture comprised 11 μl of distilled water, 10 μl of 2.5X HotMasterMix (5 Prime), 1 μl each of forward and reverse primers at 10 μM (Invitrogen), and 2 μl of template DNA. Cycle conditions were as follows: 95˚C for 2 minutes; 20 cycles of 94˚C for 30 seconds, 52˚C for 45 seconds, and 65˚C for 5 minutes; and a final extension of 65˚C for 15 minutes[2]. Following PCR, products from the triplicate assays were combined, purified using the QIAquick PCR Purification Kit (Qiagen), visualised using gel electrophoresis, and quantified via Qubit fluorometer with broad-range assay reagents (Life Technologies). A no-template control was included in each batch of PCR.

Extraction and PCR for infant samples was performed in batches of 24, with each batch containing the day-0 and -14 samples of 12 infants. Individuals were randomly assigned to extraction batches. All extractions were blinded with regard to study arm. For a small number of samples (18/240 infant samples and 10/40 adult samples), the initial PCR did not yield sufficient product for sequencing (<15 ng/μl), likely due to the presence of PCR inhibitors after DNA extraction[4]. PCR was repeated for these samples, but with the inclusion of 0.2 μl of bovine serum albumin (BSA) in the 25-μl reaction mixture. In all cases, PCR inhibition was overcome by the addition of BSA.

Purified PCR products were pooled in equimolar quantities (300 ng per sample), and sequenced in a single MiSeq run containing 240 infant samples, 40 adult samples, 12 no-template extraction controls, and one no-template PCR control. We also sequenced PCR products amplified from two mock communities obtained from BEI Resources (Manassas, USA): HM-782D, comprising genomic DNA derived from a community of 20 bacterial strains with an even rRNA operon count per organism[5]; and HM-783D, comprising genomic DNA from the same community but with an uneven ('staggered') rRNA operon count per organism[6]. Since extraction and no-template controls did not yield sufficient PCR product to be detected by Qubit, these products were added to the pool in a quantity equivalent to the lowest volume being added for a faecal DNA amplicon (personal communication, Dr Michael Cox, National Heart and Lung Institute, Imperial College London). After amplicon pooling, 1X TE buffer added to make up a total volume of 4 ml. 10 ml of 100% ethanol and 600 µl of 5 M NaCl were added to the pooled library, which was vortexed lightly and stored overnight at -20°C. The library was subsequently divided evenly into ten Eppendorf tubes, which were centrifuged for 30 minutes at full speed (14,000 *g*). Following the removal of supernatant, the pellets were washed with 800 µl of 70% ethanol, centrifuged for 5 minutes at full speed, allowed to air-dry for 10 minutes, then resuspended in 20 µl of EB buffer (Qiagen) and pooled. 100 µl of this pool was subsequently run on a 1.5% agarose gel and the band excised then purified using the QIAquick Gel Extraction Kit. The size distribution of amplicons was verified using a D1000 ScreenTape on an Agilent 2200 TapeStation. The eluted library was quantified using the KAPA Library Quantification Kit (KK4824, KAPA Biosystems) on a 7500 Fast real-time PCR system (Applied Biosystems) and diluted to a concentration of 2 nM. The library was then sequenced via Illumina MiSeq, following the 151 x 151 bp protocol described by Caporaso et al[3], with a final reaction mixture comprising 60% library DNA and 40% phi X DNA. MiSeq was performed at the MRC Clinical Sciences Centre Genomics Facility, Hammersmith Hospital, London.

Sequence processing. Forward and reverse reads from each sample were assembled into contigs using FLASH, specifying a minimum overlap size of 25 bp[7]. A length-filtering step was then performed to exclude assembled reads outside of the target range (253 ± 15 bp) using a customised Unix script (adapted from code published by Ziemann[8]). Sequences were subsequently analysed with Qiime (MacQiime version 1.8.0), using default settings unless otherwise specified. Several commands in the bioinformatic pipeline were adapted from the denovo.sh script published by Nelson et al[9]. Quality filtering was performed according to the criteria validated by Bokulich et al[10] (using the function *split_libraries_fastq.py*), but with an elevated quality score of ≤19 to define low-quality base calls. Sequences were then clustered *de novo* into operational taxonomic units (OTUs) with ≥97% nucleotide identity using uclust (*pick_otus.py*), aligned using PyNAST (*pick_rep_set.py* followed by *align_seqs.py*, using an alignment spanning the V4 region of the Greengenes sequences as reference)[11,12], and checked for chimeras using ChimeraSlayer (*parallel_identify_chimeric_seqs.py)*[13]. Taxonomic assignments

were made using naïve Bayesian Ribosomal Database Project (RDP) classifier[14] with a minimum confidence threshold of 0.8 (*parallel_assign_taxonomy_rdp.py*).

To prepare a phylogenetic tree of the V4 sequences, a separate alignment was first generated using default settings (*align_seqs.py*), enabling the Lane mask to be applied during subsequent filtering (*filter_alignment.py*)[15]. A tree was then prepared using fasttree (*make_phylogeny.py*). Unaligned or chimeric sequences were removed prior to analysis, as were any OTUs that represented <0.005% of sequences across all infant samples or <0.005% across all adult samples.

After sequence processing, we observed a minimum of 7,708 sequences per sample. To standardise sequencing depth across samples, all analyses were performed at a rarefaction depth of 7,500 sequences per sample. For the assessment of within- and between-sample diversity, we generated ten separate rarefactions at this depth and calculated mean values for OTU count, Shannon index, and between-sample Unifrac distances (unweighted and weighted). Within-sample diversity metrics were determined using the Qiime functions *multiple_rarefactions.py*, *alpha_diversity.py*, and *collate_alpha.py*, while Unifrac distances were calculated using the function *beta_diversity.py*, then visualised using principal coordinates analysis (*principal_coordinates.py*).

Preparation of V4-specific Greengenes reference files. The use of reference sequences corresponding to the 16S rRNA hypervariable region of interest has previously been shown to improve the depth and confidence of taxonomic classification during studies of the bacterial microbiota[16]. We therefore used customised reference files comprising the V4 region of the Greengenes 97%-identity OTU sequences (version 2013/08) during alignment, chimera checking, and taxonomic assignment. A V4-specific alignment was initially generated by identifying the locations of the primers 515F and 806R in the NAST-aligned Greengenes reference file, trimming the alignment 15-bp upstream and downstream of the primer locations using the function *pcr.seqs* in mothur[17], and removing common gaps using the function *filter.seqs* (analogous to the approach adopted by Nelson et al[9] to generate reference files specific to the V4–V5 region of the 16S rRNA gene). The resulting file was used as a basis for alignment and chimera checking in the pipeline outlined above. Alignment characters were removed to generate an unaligned set of V4 sequences, which was used to retrain the RDP classifier during taxonomic classification.

Results

Protocol deviations. Among the 120 infants included in the microbiota analysis, six failed to complete the study per protocol. In each of these cases, the 14-day study visit (on which oral poliovirus vaccine was administered) occurred outside of the study window (14 days ± 1 day).

Negative controls. Across the 12 extraction controls and single no-template control included in the MiSeq run, we obtained five sequences in total (0.4 sequences per sample on average), suggesting that any contamination of laboratory reagents or cross-contamination during library preparation was negligible.

Mock community profile. PCR products amplified from two mock communities (with even and staggered rRNA operon counts) were included in the MiSeq run. We restricted our attention to the mock community with an even composition during the ensuing analyses (though consistent results were obtained from the staggered community). Among the 16,860 sequences obtained from this sample after quality filtering, we excluded OTUs with a relative abundance of <0.1%, then matched the remaining OTUs (n = 18) to their corresponding taxa in the mock community based on genus-level taxonomic classifications or (where the mock community contained multiple strains in the same genus) using BLAST [18]. The relative abundances of these taxa were generally consistent with their expected values (Supplementary Fig. 1). Although we observed no sequences corresponding to the strain *Propionibacterium acnes* and under-representation of strains in the genera *Enterococcus, Deinococcus,* and *Rhodobacter*, these findings are consistent with the biases documented in previous studies using the primers 515F and 806R[9].

Impact of non-intervention antibiotics. As noted in the main text, among the 114 infants included in our microbiota analyses who completed the study per protocol, 28 (25%) used antibiotics in the month before enrolment (which was delayed until after infants had stopped taking antibiotics), and 10 (9%) took antibiotics other than the study intervention between days 0 and 14. The proportion of infants exposed to non-intervention antibiotics did not differ significantly according to study arm before enrolment (12/56 [21%] vs 16/58 [28%] in recipients of azithromycin and placebo, respectively; Fisher's test, p = 0.517) or between days 0 and 14 (3/56 [5%] vs 7/58 [12%]; Fisher's test, p = 0.322).

Our ability to determine the impact of non-intervention antibiotics on the bacterial microbiota was constrained by limitations in sample size as well as the diversity of antibiotic regimens to which infants were exposed. Nonetheless, pre-enrolment antibiotic exposure was associated with a significant decline in the number of OTUs observed at day 0 (66.7 ± 14.9 vs 74.0 ± 13.9 in exposed and non-exposed infants, respectively; linear regression, p = 0.009; Supplementary Fig. 6A) and – analogous to the observed effect of azithromycin – a non-significant decline in Shannon index (2.6 ± 0.5 vs 2.8 ± 0.5; linear regression, p = 0.106; Supplementary Fig. 6C). Prior antibiotic exposure was also linked with a decrease in microbiota age at day 0 based on unweighted Unifrac distances from adults (0.837 ± 0.048 vs 0.814 ± 0.044 in exposed and non-exposed infants; linear regression, p = 0.012) but not weighted distances (0.606 ± 0.044 vs 0.597 ± 0.044; linear regression, p = 0.367). At day 14, we observed no significant differences in these metrics according to pre-enrolment antibiotic exposure status (p values

>0.05 for all comparisons), implying a relatively swift normalisation of microbiota composition after the cessation of antibiotic use. To further explore this possibility, we compared OTU count and Shannon index between day-0 and day-14 samples within each study arm among infants exposed to antibiotics before enrolment but no other non-intervention antibiotics. Although no longitudinal comparisons were significant within this small subset of infants (Wilcoxon's signed rank test, p values >0.05), microbiota diversity tended to increase between days 0 and 14 among placebo recipients but not azithromycin recipients (Supplementary Fig. 6B and 6D).

In contrast to the effect of azithromycin, we did not observe any significant clustering of samples at days 0 or 14 according to pre-enrolment antibiotic exposure (adonis, p values >0.05 for unweighted and weighted Unifrac distances). Likewise, few discrepancies in taxon relative abundance were observed between these groups (Supplementary Table 4), possibly reflecting the contrasting effects of different antibiotic regimens on microbiota composition.

References

1 Yuan, S., Cohen, D. B., Ravel, J., Abdo, Z. & Forney, L. J. Evaluation of methods for the extraction and purification of DNA from the human microbiome. *PloS One* 7, e33865 (2012).

2 Dinh, D. M. *et al.* Longitudinal Analysis of the Intestinal Microbiota in Persistently Stunted Young Children in South India. *PloS One* **11**, e0155405 (2016).

3 Caporaso, J. G. *et al.* Ultra-high-throughput microbial community analysis on the Illumina HiSeq and MiSeq platforms. *ISME J.* **6**, 1621-1624 (2012).

4 Schrader, C., Schielke, A., Ellerbroek, L. & Johne, R. PCR inhibitors - occurrence, properties and removal. *J. Appl. Microbiol.* **113**, 1014-1026 (2012).

5 BEI Resources. Product information sheet for HM-782D. Available from: https://www.beiresources.org/ProductInformationSheet/tabid/784/Default.aspx?doc=38933.pdf [Last accessed: 03 June 2016].

6 BEI Resources. Product information sheet for HM-783D. Available from: https://www.beiresources.org/ProductInformationSheet/tabid/784/Default.aspx?doc=39237.pdf [Last accessed: 03 June 2016].

7 Magoc, T. & Salzberg, S. L. FLASH: fast length adjustment of short reads to improve genome assemblies. *Bioinformatics* **27**, 2957-2963 (2011).

8 Ziemann, M. Fastq to tabular - filter reads on length. http://genomespot.blogspot.co.uk/2012/12/fastq-to-tabular-filter-reads-on-length.html [Accessed 22 April 2016].

9 Nelson, M. C., Morrison, H. G., Benjamino, J., Grim, S. L. & Graf, J. Analysis, optimization and verification of Illumina-generated 16S rRNA gene amplicon surveys. *PloS One* **9**, e94249 (2014).

10 Bokulich, N. A. *et al.* Quality-filtering vastly improves diversity estimates from Illumina amplicon sequencing. *Nat. Methods* **10**, 57-59 (2013).

11 DeSantis, T. Z., Jr. *et al.* NAST: a multiple sequence alignment server for comparative analysis of 16S rRNA genes. *Nucleic Acids Res.* **34**, W394-399 (2006).

12 Caporaso, J. G. *et al.* PyNAST: a flexible tool for aligning sequences to a template alignment. *Bioinformatics* **26**, 266-267 (2010).

13 Haas, B. J. *et al.* Chimeric 16S rRNA sequence formation and detection in Sanger and 454-pyrosequenced PCR amplicons. *Genome Res.* **21**, 494-504 (2011).

14 Wang, Q., Garrity, G. M., Tiedje, J. M. & Cole, J. R. Naive Bayesian classifier for rapid assignment of rRNA sequences into the new bacterial taxonomy. *Appl. Environ. Microbiol.* **73**, 5261-5267 (2007).

15 Lane, D. J. 16S/23S rRNA sequencing. In: *Nucleic Acid Techniques in Bacterial Systematics* (eds Goodfellow M. & Stackebrandt E.), 115-175 (1991).

16 Werner, J. J. *et al.* Impact of training sets on classification of high-throughput bacterial 16s rRNA gene surveys. *ISME J.* **6**, 94-103 (2012).

17 Schloss, P. D. *et al.* Introducing mothur: open-source, platform-independent, community-supported software for describing and comparing microbial communities. *Appl. Environ. Microbiol.* **75**, 7537-7541 (2009).

18 Johnson, M. *et al.* NCBI BLAST: a better web interface. *Nucleic Acids Res.* **36**, W5-9 (2008).
